# Effect of Financial Education and Coaching on Economic Stability in Low Income, Single Mother Families

**DOI:** 10.1007/s10935-026-00909-7

**Published:** 2026-04-20

**Authors:** Nicole D. White, Julie C. Kalkowski, Ryan W. Walters, Kathy A. Flecky, Lisa L. Black, Jennifer A. Furze, Julie A. Peterson, Lorraine M. Rusch, Ann M. Ryan Haddad, Kevin Fuji

**Affiliations:** 1 Creighton University School of Pharmacy and Health Professions, 2500 California Plaza, Omaha, NE 68178 USA; 2 Financial Hope Collaborative, Creighton University, Omaha, USA; 3 Creighton University School of Medicine, Omaha, USA; 4 Creighton University College of Nursing, Omaha, USA

**Keywords:** Financial education, Financial coaching, Financial stress, Economic stability

## Abstract

Up to 85% of health is governed by social determinants such as economic stability. By supporting healthy financial behavior changes to increase economic stability, the Financial Success Program (FSP) is a modality that also addresses the limitations of other health interventions in single mother, low-income households. The novel FSP financial education/coaching intervention has previously been shown to reduce financial strain and perceived effects of financial strain on health, improve relationships, reduce smoking and increase healthcare access. This manuscript reports the financial and economic stability outcomes of the first randomized controlled trial to assess the impact of the FSP financial education/coaching model in 345 single mother low-income households. FSP participants experienced the following positive economic outcomes compared with control participants: significantly greater increases in mean salary and household income; greater increases in savings; larger reductions in the inability to save money; larger reductions in shut off notices and utility balances; greater increases in paying bills on time, reductions in partial payments, late fees, and overdrawn bank accounts; greater increases in effective monthly cashflow management and decreases in counterproductive financial behaviors. FSP significantly built participants’ capacity to increase income, emergency savings and retirement savings. The FSP model fosters beneficial financial behaviors while diminishing detrimental financial behaviors, as compared to usual care. By mitigating financial stress, individuals graduating from the FSP increase their capability to plan ahead and think more clearly about financial decisions to improve economic stability, and ultimately overall health.

## Background

It is proposed that up to 85% of health is determined by unmet social needs, or social determinants of health (SDOH) (Fenton, 2011). Economic stability is a SDOH and an important driver for the health disparities observed in low-income individuals (Kessler & Clearly, 1980, Metlock et al., 2024; Seeman & Crimmins, 2001; Turner et al., 1995). The relationship between income and health falls on a socioeconomic spectrum and the gradient appears to be a stronger predictor of health than race or ethnicity (Woolf et al., 2015). In fact, the County Health Rankings Model assigns social and economic factors a weight of 40% for overall health, half of which is comprised by income and employment (Hood et al., 2016). Social Determinants of Health are considered a public health priority. In the United States, the Healthy People, 2030 framework emphasize SDOH as an approach to addressing health equity and disease prevention ((U.S. Department of Health and Human Services, 2026).

Financial stress has been operationalized as “the physical or mental health symptoms that arise from having difficulty meeting basic needs, difficulty paying bills, and money leftover at the end of the month” (Friedline et al., 2020). Financial stress has been implicated in cardiovascular disease risk (Eaker et al., 1992; Ma et al., 2025; Rosengren et al., 2004). A recent study found that chronic financial stress was associated with elevated risks of stroke, myocardial infarction, and heart failure (Ma et al., 2025). A review of literature from 2010–2019 indicated that women have poorer physical and mental health because of financial stress when compared to males. Financial stress has historically impacted a disproportionate number of women and minorities, especially African Americans. Data from the 2017 Diary of Consumer Payment Choice indicated a mean savings of $2,173 for African Americans versus $13,564 for Whites (Stavins, 2020). Likewise, more African Americans are unable to save at all as compared to Whites (39.9% versus 22.4%) (Stavins, 2020). Economic stress is on the rise in the United States. In 2019, 46% of Americans reported economic stress which increased to 63% in 2020 (presumably due to COVID-19), and increased further to 75% in 2025 (American Psychological Association, 2020; American Psychological Association, 2025). Not only did COVID-19-associated job loss impact women and low-income individuals more than others, the economic fallout impacted these individuals disproportionately, too (Parker et al., 2020). Compared to middle-income households, low-income households were more likely to report using money from savings or retirement to pay bills, had trouble paying bills, or had trouble paying rent or mortgage while women were more likely to report trouble paying bills compared to men (Parker et al., 2020).

Persons under financial strain are more likely to engage in smoking, alcohol consumption, overspending, poor diet, and reduced exercise (Probst et al., 2020; Vohs, 2013). This can be explained by the limited-resource model of self-control that suggests self-control is a limited resource and depletion leads to decision fatigue (Mullainathan & Shafir, 2013). Because of less money, food, and bandwidth, low-income individuals make more difficult decisions more frequently than individuals with higher incomes. This increased behavior regulation depletes mental function, exhausts self-control, and leads to behaviors that are harmful to financial and physical health. Such “bandwidth tax” (Mullainathan & Shafir, 2013, p. 39) is also why interventions to promote health in low income individuals are often unsuccessful (Mullainathan & Shafir, 2013; Pearlin et al., 1981). A modality to address the limitations of other health interventions in single mother, low-income, households is the Financial Success Program (FSP).

The FSP is a financial education and coaching intervention to improve health through action on the SDOH, economic stability. The FSP has been operating since 2009 at Creighton University in Omaha, Nebraska and has a consistent track record of promoting durable financial behavior change, reducing financial stress, and improving quality of life (Packard et. al., 2015; White et. al., 2018). The FSP has recently been shown to reduce financial strain and the perceived effects of financial strain on health, as well as decrease the avoidance of healthcare due to cost (White et al., 2021a, 2021b). Participants completing the FSP also experienced significant reductions in smoking superior to the increase observed in the control group. This analysis evaluates the impact of the FSP on financial behaviors and economic stability in single-mother low-income households. By mitigating financial stress and improving economic stability, graduates improve capability to plan ahead and think clearly about financial decisions. The research hypothesis proposes that the financial education and coaching intervention will increase productive financial behaviors including on-time bill pay and contributing to savings and retirement, while reducing detrimental financial behaviors such as paying late fees, overdrawn bank accounts and maxing out credit cards.

## Methods

This randomized controlled trial was conducted from 2017–2020 and enrolled 345 single mothers 19–55 years. The FSP is an established program who receives referrals from a wide net of non-profits/community-based organizations (pantries, shelters, social service organizations, etc.). The program has also had a long-standing waitlist to participate. Radio ads were used to supplement recruitment by referral. Women were enrolled from the established wait-list, referred by community partners, or referred by word of mouth from past participants. Participants were included if they were employed, earned less than 200% of the US Federal Poverty Guideline, and spoke English and/or Spanish. Women were excluded if they were pregnant, planning to become pregnant, abusing alcohol or illicit drugs, or in a domestic violence situation. The study was approved by the Creighton University IRB (InfoEd record 1,011,656) and all participants provided written informed consent.

Participants were randomized on a rolling basis to either the FSP which included nine weeks of financial education plus 12 months of one-on-one financial coaching or control. An equal allocation randomization schedule was created; however, because the FSP utilizes word of mouth recruitment, participants enrolled upon referral were assigned to the same group (FSP or control) to which the referrer was randomized. Although a threat to internal validity, this limited contamination of control participants who might receive indirect financial education/coaching from the referrer. A total of 20 women referred one or more acquaintances, resulting in 32 enrolling via referral, 27 of whom received the intervention. Referral was accounted for in the statistical analysis whenever possible. Women randomized to the control group, did not receive the FSP intervention, or any other specified intervention during the twelve-month follow-up period. All women randomized to the control group, were offered an opportunity to complete the FSP following study completion to comply with ethical standards.

## Intervention

The FSP's year-long intervention helps families feel connected and supported by normalizing and reframing their financial circumstances to envision a better future. Participants are encouraged to address immediate financial issues, learn and practice skills and behavior changes, and develop decision making strategies to foster financial confidence. The model's multiple interactive elements provide ongoing group support; year-long one-on-one coaching; and other value-added components that builds financial capacity and confidence. The FSP concentrates on three core components: an outstanding trainer, financial coaching, and an easy to use money management system. Program strategies include monthly cashflow management, interactive education, validating emotions, values clarification, pros and cons of making changes, and forming positive habits to take effective action to support financial well-being (Prochaska et al., 2015). Program participants receive nine weeks of in-person group financial education aimed at addressing the immediate financial needs of low- to moderate- income households. Each weekly group session lasts three hours and includes childcare and dinner for the participant and their children. Topics covered include the psychology of money, predatory lending, credit reports, level payment plans, collections, insurance, retirement, banking and savings.

Following the group educational classes, each participant was paired with a financial coach, who worked with them one-on-one for the remainder of the year. Coaches were instructed to meet with participants at least once per month. Coaching visits were conducted in-person, over the phone and through text/email, based on the participant’s preference. A number of different trainers and coaches were utilized during the intervention. Trainers are not considered financial experts, but are selected for their ability to engage participants. Trainers receive a standardized training and all curricular materials (powerpoint presentations, budgeting tool, etc.) to teach the group classes. Financial coaches are also not considered financial experts, but are selected for their ability to support and encourage program participants. Many trainers are former graduates of the FSP. The program was offered in English and Spanish and the detailed curriculum was previously published (White et al., 2015).

## Data Collection

Study recruitment and enrollment took place between April 2017 and September 2019. Written informed consent was obtained prior to intervention and participants completed study visits with investigators at baseline and 12 months. Due to COVID-19, 12-month visits for participants finishing the study after March 2020 were completed by telephone, with an IRB-approved amendment. Demographics were collected at the initial and 12-month study visit. Participants were administered a program-developed financial history survey which was also administered at baseline and at the 12-month study visit. This survey assessed salary, expenses, savings, loans, housing and utility costs, as well as financial behaviors such as on-time bill payments, setting financial goals and budgeting. The frequency and mode (in-person visit, phone call, text/email) of coaching visits was collected by a phone call to each participant on a monthly basis.

### Statistics

All descriptive statistics were stratified by intervention and control cohorts. Depending on data distribution, continuous variables are presented as mean and standard deviation or median and interquartile range. Categorical variables are presented as frequency count and percent. Between-group differences at baseline and follow-up were compared using independent-samples *t*-test or the Mann–Whitney test for continuous variables, and chi-square test or Fisher’s exact test for categorical variables. Within-group change from baseline was evaluated using paired-samples *t*-test or Wilcoxon signed-ranks test for continuous variables, and McNemar’s test for categorical variables. Whether within-group change differed between the intervention and control groups was evaluated using a time-by-group interaction effect estimated via a mixed-effects linear, lognormal, logistic, or proportional-odds regression model with the choice of statistical model based on each outcome’s scale of measurement (e.g., continuous, binary) and distributional assumptions (e.g., normal, skewed with non-constant variance); a mixed-effects model was required to account for the correlation inherent to outcome measurements observed from the same woman. All analyses were conducted using SAS v. 9.2 (SAS Institute Inc., Cary, NC) using both intent-to-treat (ITT) and per-protocol approaches (PP) with *p* ≤ 0.05 was used to indicate statistical significance. Both analyses included all participants randomized to control. The ITT analysis included all participants randomized to the FSP regardless of weeks of completed financial education (including none), whereas the PP analysis included participants randomized to the FSP who completed at least 7 of the 9 financial education classes and graduated (i.e., approximately 80% of the intervention).

## Results

### Demographics

**Fig. 1:**
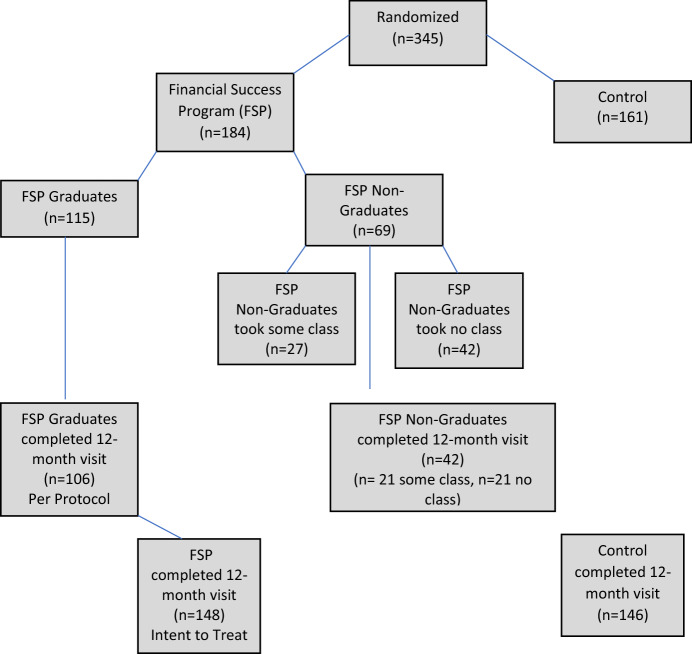
Randomization scheme

Of the 345 participants enrolled (Fig. 1), 294 returned for their 12-month visit and 51 were lost to follow-up.

Of the 184 women randomized to the FSP, 148 returned for their 12-month visit and were included in the ITT analysis while 115 (62.5%) graduated and 106 graduates returned for their 12-month visit and were included in the per protocol (PP) analysis (Table 1).

**Table 1:** Baseline demographics

	Control^a^ (n = 146)	Intervention Intent to Treat (ITT)^b^ (n = 148)	*p* value (Versus Control)	Intervention Per Protocol (PP)^c^ (n = 106)	*p* Value (Versus Control)
Age (Median [interquartile range])	35.1 + 7.8	34.6 + 7.6	0.488	34.9 + 7.2	0.485
Spanish Speaking (%)	6.8	10.3	0.251	13.0	0.082
Race/Ethnicity					
Black/African American (%)	54.0	54.9	0.874	54.8	0.903
Non-Hispanic White (%)	29.8	21.2	0.066	18.3	0.029*
Latina/Hispanic (%)	13.0	16.3	0.395	19.1	0.169
Multiple Race/Ethnicity (%)	3.1	4.4	0.546	4.4	0.596
Other (%)	0	3.3	0.032*	3.5	0.029*
Highest Education Achieved					
Some High School (%)	6.3	7.6	0.622	7.8	0.611
High School Graduate (%)	8.8	14.1	0.121	11.3	0.483
Some College (%)	48.1	44.0	0.446	48.7	0.926
College Graduate (%)	36.9	33.7	0.538	32.2	0.420
Marital Status					
Single (%)	68.9	71.2	0.648	71.3	0.673
Married (%)	1.2	0	0.217	0	0.512
Divorced (%)	21.1	16.9	0.312	15.7	0.252
Separated (%)	6.2	10.9	0.126	11.3	0.131
Widowed (%)	3.1	0.5	0.102	0.9	0.209
Number of Children Living with Mother (Median [interquartile range])	2 [1—3]	2 [1—3]	0.036*	2 [1—3]	0.110

^*^P < 0.05

^a^Included participants who were randomized to the control group

^b^Included participants who were randomized to financial education/coaching who completed 9 weeks of financial education as well as those who were randomized to financial education/coaching who did not complete 9 weeks of financial education

^c^Included participants who were randomized to financial education/coaching who completed 9 weeks of financial education

Half of the 42 participants in the ITT analysis who were not included in the PP analysis completed some components of the FSP. Reasons for starting the FSP and not graduating are varied, but include sudden life events, family emergency and job loss. Most participants were Black/African American with some college experience. There were significantly less non-Hispanic, White women in the PP analysis (18.3%) as compared to the control group (29.8%). This suggests a significant proportion of non-Hispanic White women randomized to the FSP either did not start or did not graduate.

## Financial Outcomes

FSP participants in the PP analysis interacted with their financial coach face-to-face (89.3%), via phone or text (88.4%), and/or via email (59.8%). Face-to-face meetings occurred at a median rate of 0.5 times per month [0.3–0.7], phone calls or texts 1.2 times per month [0.6–2.0] and emails 0.5 times per month [0.2–1.0]. Results for the ITT analysis, which included 148 participants randomized to FSP, are summarized in Table 2.

**Table 2:** Financial Outcomes – Intent to Treat

					*p* Values				
	Control^a^ (n = 146)		Intervention Intent to Treat (ITT)^b^ (n = 148)		Within Group		Between Group		Interaction
	Baseline	12 Months	Baseline	12 Months	Control	ITT	Baseline	12 Months	
Salary and Promotion									
Mean Salary (thousands) ± SD	24.3 ± 10.4	26.3 ± 12.1	23.9 ± 9.5	29.3 ± 13.7	0.002*	< 0.001*	0.699	0.047*	0.009*
Mean household Income (thousands) ± SD	26.4 ± 10.6	29.0 ± 13.8	25.9 ± 9.7	32.2 ± 16.1	0.004*	< 0.001*	0.653	0.064	0.019*
Mean Monthly Household Expenses (thousands) ± SD	1.7 ± 0.8	1.7 ± 0.7	1.6 ± 1.0	1.5 ± 0.8	0.237	0.106	0.705	0.014*	0.066
Living Expenses Covered in Past Year (%)	19.9	37.0	28.8	56.8	< 0.001*	< 0.001*	0.055	< 0.001*	0.242
Promotion Received in Past Year (%)	18.6	`18.5	19.6	28.4	0.732	0.020*	0.826	0.046*	0.154
Job Classification in Past Year									
Professional (%)	42.2	36.3	39.1	39.2	0.103	0.201	0.558	0.610	0.527
Clerical (%)	19.3	20.6	19.6	12.8	1.00	0.008*	0.942	0.076	0.043*
Administrative (%)	13.0	14.4	10.3	16.9	1.00	0.046*	0.432	0.554	0.182
Sales (%)	11.2	7.5	14.7	10.8	0.366	0.763	0.336	0.331	0.503
Unskilled (%)	11.2	9.6	16.3	16.9	0.527	0.808	0.170	0.065	0.508
Technical (%)	3.7	4.1	1.6	2.7	0.564	0.317	0.314	0.540	0.498
Civil Service (%)	3.1	3.4	1.6	2.0	1.00	0.564	0.481	0.500	0.737
Savings and Retirement in Past Year									
Not Able to Save Money (%)	62.7	52.7	54.9	24.3	0.039*	< 0.001*	0.140	< 0.001*	0.003*
Saving Money Regularly (%)	5.6	12.3	6.5	34.5	0.033*	< 0.001*	0.718	< 0.001*	0.026*
Frequency of Contributing to Retirement in Past Year									
Never (%)	55.3	48.0	67.9	51.4	0.117	0.003*	0.016*	0.559	0.149
Seldom (%)	5.6	9.6	5.4	5.4	0.071	1.000	0.950	0.173	0.318
Sometimes (%)	8.1	5.5	4.4	4.1	0.491	0.593	0.149	0.566	0.635
Frequently (%)	8.1	11.6	4.4	6.1	0.394	0.808	0.149	0.093	0.925
Always (%)	24.7	23.0	33.1	18.5	1.000	0.002*	0.302	0.110	0.017*
Housing									
Housing to Income Ratio ≥ 30% (%)	53.4	48.6	46.2	29.3	0.114	0.001*	0.181	< 0.001*	0.069
Utilities in Past Year									
On Utility Level Payment Plan (*)	28.5	37.7	37.4	49.3	0.064	0.008*	0.083	0.044*	0.724
Past Due Balance on Utilities (%)	26.1	22.6	24.5	10.8	0.238	< 0.001*	0.728	0.007*	0.036*
Received Shut Off Notice in Past Year (%)	70.2	63.0	71.2	50.0	0.023*	< 0.001*	0.837	0.024*	0.017*
Number of Shut Off Notices in Past Year (Median [interquartile range])	3 [2—5]	3 [2—5]	3 [2—5]	3 [1—5]	0.772	< 0.001*	0.867	0.003*	< 0.001*
Bill Payments in Past Year									
On Time (%)	52.8	58.2	45.1	65.5	0.123	< 0.001*	0.154	0.196	0.033*
Partial Payment (%)	63.4	58.2	63.6	39.2	0.123	< 0.001*	0.964	0.001*	0.005*
Ignore Bills (%)	41.0	30.8	29.4	13.5	0.014*	< 0.001*	0.024*	< 0.001*	0.085
Bills Have Late Fees (%)	86.3	82.2	88.6	66.9	0.201	< 0.001*	0.528	0.003*	0.003*
Monthly Bills with Late Fees (Median [interquartile range])	2 [2—3]	2 [2—3]	2 [2—3]	2 [1—3]	0.135	< 0.001*	0.936	0.171	0.176
Experienced Overdrawn Bank Account in Past Year (%)	72.1	64.4	70.1	50.0	0.096	< 0.001*	0.692	0.013*	0.046*
Number of Times Overdrawn Bank Account in Past Year (Median [interquartile range])	3 [2—5]	3 [2—5]	3 [2—5]	3 [1—5]	0.416	0.173	0.284	0.228	0.640
Cash Flow Management in Past Year									
Writes Down and Prioritizes Financial Goals (%)	43.9	47.9	43.8	71.4	0.602	< 0.001*	0.982	< 0.001*	< 0.001*
Uses Written Budget (%)	28.8	38.9	26.8	57.1	0.009*	< 0.001*	0.684	0.002*	0.004*
Reviews Spending (%)	31.0	46.9	32.2	64.9	0.001*	< 0.001*	0.812	0.002*	0.017*
Detrimental Financial Behaviors in Past Year									
Pay Day Lender Use in Past Year (%)	26.1	23.3	26.1	14.9	0.578	0.002*	1.000	0.066	0.057
Number of Times Pay Day Lender Use in Past Year (Median [interquartile range])	3 [1—5]	3 [2—5]	2 [1—4]	2 [1—4]	0.961	0.688	0.651	0.093	0.190
Frequency of Borrowing Money in Past Year									
Never (%)	10.6	23.3	13.6	35.1	< 0.001*	< 0.001*	0.391	0.026*	0.463
Seldom (%)	18.0	20.6	20.7	30.4	0.537	0.096	0.536	0.053	0.352
Sometimes (%)	37.3	34.9	44.0	21.0	0.606	< 0.001*	0.203	0.008*	0.003*
Frequently (%)	27.3	14.4	15.2	10.8	0.003*	0.317	0.006*	0.336	0.280
Always (%)	6.8	7.5	6.5	2.7	1.000	0.096	0.908	0.060	0.134
Frequency of Maxing Out Credit Card in Past Year									
Never (%)	51.6	52.7	47.8	60.8	1.000	< 0.001*	0.490	0.163	0.015*
Seldom (%)	5.0	12.3	4.9	13.5	0.041*	0.014*	0.974	0.762	0.839
Sometimes (%)	14.3	11.0	10.9	10.1	0.336	0.480	0.338	0.818	0.662
Frequently (%)	8.7	13.0	16.9	9.5	0.103	0.016*	0.025*	0.334	0.025*
Always (%)	20.5	10.3	19.6	6.1	0.006*	0.002*	0.829	0.189	0.296

^*^P < 0.05

^a^Included participants who were randomized to the control group

^b^Included participants who were randomized to financial education/coaching who completed 9 weeks of financial education as well as those who were randomized to financial education/coaching who did not complete 9 weeks of financial education

Results for the PP analysis, which included 106 participants that completed seven or more of the nine total financial education classes, are summarized in Table 3.

**Table 3:** Financial Outcomes – Per Protocol

					*p* Values				
	Control^a^ (n = 146)		Per Protocol (PP)^b^ (n = 106)		Within Group		Between Group		Interaction
	Baseline	12 Months	Baseline	12 Months	Control	PP	Baseline	12 Months	
Salary and Promotion									
Mean Salary (thousands) ± SD	24.3 ± 10.4	26.3 ± 12.1	24.2 ± 9.3	29.1 ± 13.4	0.002*	< 0.001*	0.947	0.084	0.029*
Mean household Income (thousands) ± SD	26.4 ± 10.6	29.0 ± 13.8	26.2 ± 9.8	32.5 ± 16.9	0.004*	< 0.001*	0.881	0.081	0.035*
Mean Monthly Household Expenses (thousands) ± SD	1.7 ± 0.8	1.7 ± 0.7	1.6 ± 0.7	1.6 ± 0.8	0.237	0.488	0.389	0.122	0.256
Living Expenses Covered in Past Year (%)	19.9	37.0	33.0	63.3	< 0.001*	< 0.001*	0.013*	< 0.001*	0.234
Promotion Received in Past Year (%)	18.6	18.5	21.7	29.3	0.732	0.144	0.524	0.045*	0.314
Job Classification in Past Year									
Professional (%)	42.2	36.3	35.7	35.9	0.103	0.796	0.270	0.941	0.323
Clerical (%)	19.3	20.6	22.6	14.2	1.00	0.002*	0.497	0.191	0.022*
Administrative (%)	13.0	14.4	11.3	17.0	1.00	0.058	0.665	0.574	0.259
Sales (%)	11.2	7.5	12.2	9.4	0.366	1.00	0.799	0.590	0.594
Unskilled (%)	11.2	9.6	17.4	20.8	0.527	0.593	0.140	0.012*	0.332
Technical (%)	3.7	4.1	1.7	2.8	0.564	0.564	0.332	0.738	0.672
Civil Service (%)	3.1	3.4	2.6	2.8	1.00	0.564	1.00	1.00	0.803
Savings and Retirement in Past Year									
Not Able to Save Money (%)	62.7	52.7	53.9	16.0	0.039*	< 0.001*	0.142	< 0.001*	< 0.001*
Saving Money Regularly (%)	5.6	12.3	4.4	39.6	0.033*	< 0.001*	0.643	< 0.001*	0.004*
Frequency of Contributing to Retirement in Past Year									
Never (%)	55.3	48.0	59.1	47.2	0.117	0.041*	0.524	0.903	0.513
Seldom (%)	5.6	9.6	7.0	6.6	0.071	1.000	0.642	0.397	0.302
Sometimes (%)	8.1	5.5	5.2	4.7	0.491	0.763	0.355	0.787	0.689
Frequently (%)	8.1	11.6	5.2	3.8	0.394	0.527	0.355	0.026*	0.315
Always (%)	24.7	23.0	23.5	37.7	1.000	0.009*	0.923	0.026*	0.045*
Housing									
Housing to Income Ratio ≥ 30% (%)	53.4	48.6	44.4	30.5	0.114	0.005*	0.137	0.004*	0.169
Utilities in Past Year									
On Utility Level Payment Plan (*)	28.5	37.7	36.5	51.9	0.064	0.009*	0.159	0.025	0.474
Past Due Balance on Utilities (%)	26.1	22.6	23.5	8.5	0.238	0.002*	0.622	0.003*	0.031*
Received Shut Off Notice in Past Year (%)	70.2	63.0	67.8	42.5	0.023*	< 0.001*	0.675	0.001*	0.007*
Number of Shut Off Notices in Past Year (Median [interquartile range])	3 [2—5]	3 [2—5]	3 [2—5]	2 [1—3]	0.772	< 0.001*	0.980	< 0.001*	< 0.001*
Bill Payments in Past Year									
On Time (%)	52.8	58.2	48.7	70.8	0.123	< 0.001*	0.502	0.041	0.021*
Partial Payment (%)	63.4	58.2	65.2	33.0	0.123	< 0.001*	0.750	< 0.001*	< 0.001*
Ignore Bills (%)	41.0	30.8	31.3	7.6	0.014	< 0.001*	0.100	< 0.001*	0.003*
Bills Have Late Fees (%)	86.3	82.2	88.7	61.3	0.201	< 0.001*	0.561	< 0.001*	0.001*
Monthly Bills with Late Fees (Median [interquartile range])	2 [2—3]	2 [2—3]	2 [1—3]	2 [1—3]	0.135	< 0.001*	0.635	0.015*	0.033*
Experienced Overdrawn Bank Account in Past Year (%)	72.1	64.4	71.3	48.1	0.096	< 0.001*	0.892	0.010*	0.026*
Number of Times Overdrawn Bank Account in Past Year (Median [interquartile range])	3 [2—5]	3 [2—5]	3 [2—5]	3 [1—5]	0.416	0.189	0.347	0.225	0.451
Cash Flow Management in Past Year									
Writes Down and Prioritizes Financial Goals (%)	43.9	47.9	39.1	78.1	0.602	< 0.001*	0.437	< 0.001*	< 0.001*
Uses Written Budget (%)	28.8	38.9	25.2	66.7	0.009*	< 0.001*	0.517	< 0.001*	< 0.001*
Reviews Spending (%)	31.0	46.9	27.7	70.8	0.001*	< 0.001*	0.554	< 0.001*	< 0.001*
Detrimental Financial Behaviors in Past Year									
Pay Day Lender Use in Past Year (%)	26.1	23.3	25.2	13.2	0.578	0.007*	0.871	0.044*	0.056
Number of Times Pay Day Lender Use in Past Year (Median [interquartile range])	3 [1—5]	3 [2—5]	2 [1—3]	2 [1—4]	0.961	0.281	0.366	0.073	0.260
Frequency of Borrowing Money in Past Year									
Never (%)	10.6	23.3	13.9	40.6	< 0.001*	< 0.001*	0.397	0.003*	0.228
Seldom (%)	18.0	20.6	25.2	28.3	0.537	0.622	0.148	0.154	0.984
Sometimes (%)	37.3	34.9	43.5	19.8	0.606	< 0.001*	0.299	0.009*	0.006*
Frequently (%)	27.3	14.4	13.0	9.4	0.003*	0.346	0.004*	0.238	0.362
Always (%)	6.8	7.5	4.4	1.9	1.000	0.180	0.384	0.045*	0.263
Frequency of Maxing Out Credit Card in Past Year									
Never (%)	51.6	52.7	45.2	57.6	1.000	0.002*	0.299	0.449	0.042*
Seldom (%)	5.0	12.3	5.2	16.0	0.041*	0.012*	0.926	0.401	0.696
Sometimes (%)	14.3	11.0	10.4	12.3	0.336	0.835	0.343	0.749	0.368
Frequently (%)	8.7	13.0	20.9	10.4	0.103	0.029*	0.004*	0.524	0.016*
Always (%)	20.5	10.3	18.3	3.8	0.006*	< 0.001*	0.644	0.054	0.103

^*^P < 0.05

^a^Included participants who randomized to the control group

^b^Included participants who were randomized to financial education/coaching who completed 9 weeks of financial education

### Salary and Promotion

Both cohorts experienced significant mean annual salary increases, but FSP participants experienced significantly larger increases in both mean salary (ITT:* p* = 0.009; PP: *p* = 0.029) and household income (ITT: *p* = 0.019; PP: *p* = 0.035) compared to control. The mean salary increase for FSP participants in the ITT analysis was $5.4 K (23%),$4.9 K (20%) for PP, and $2.6 K (9%) for control. At baseline just 19.9% of women in the control group, 28.8% of the women in the ITT, and 33% of women in the PP group reported the ability to cover living expenses. At the 12-month study visit, this increased to 37.0%, 56.8% and 66.3% for control, ITT, and PP groups, respectively. While all cohorts reported significant increases in ability to cover living expenses, the difference between groups was insignificant.

There were no significant changes in self-reported job classification for control participants whereas FSP participants reported a significant reduction in clerical positions (ITT: −6.8%, *p* = 0.043; PP: −8.4%, *p* = 0.022). In the ITT analysis there was a 6.6% increase (*p* = 0.046 versus baseline) in the rate of FSP participants reporting administrative positions but the interaction with the control group was not significant *(p* = 0.182*).* A similar trend in administrative positions was seen in the PP analysis. In the ITT, but not PP analysis, FSP participants reported a significant increase in job promotions (+ 8.8%, *p* = 0.020 versus baseline), but the increase was not significantly greater than control.

### Savings and Retirement

Both cohorts reported significant reductions in inability to save money compared to baseline. The reduction in the FSP cohort was significantly greater than that experienced by the control group (Control: −10.0%; ITT: −30.6%, *p* = 0.003; PP: −37.9%, *p* < 0.001). Similarly, both cohorts reported significant increases in rate of saving regularly. The increase in the FSP cohort was significantly higher than experienced by the control group (Control: + 6.7%; ITT: + 28%, *p* = 0.026; PP: + 35.2%, *p* = 0.004). FSP participants in the ITT analysis reported a significant reduction in the rate of always contributing to retirement (−14.6%, *p* = 0.002 versus baseline) which was significantly greater than control group (−1.7%, *p* = 0.017). Conversely, FSP participants in the PP analysis reported a significant increase in the rate of always contributing to retirement (+ 14.2%, *p* = 0.009 versus baseline) which was significantly greater than control (−1.7%, *p* = 0.045).

### Housing

FSP participants experienced significant reductions in the percentage of those spending ≥ 30% of gross income on rent or mortgage (ITT: −16.9%, *p* = 0.001; PP: −13.9%, *p* = 0.005, versus baseline), but this decrease was not significantly greater than control (−4.8%; ITT: p = 0.069; PP:* p* = 0.169).

### Utilities

Level-pay utility plans allow customers to pay a consistent monthly utility bill, regardless of seasonal fluctuations. The monthly amount is estimated by reviewing usage history from the previous 12-months. This allows the homeowner to budget more effectively. FSP participants reported a significant increase in utility level payment plan participation (ITT: + 11.9%, *p* = 0.008; PP: + 15.4%, *p* = 0.009), but this increase was not significantly greater than control (+ 9.2%; ITT: *p* = 0.724, PP: *p* = 0.474). FSP participants reported significant reductions in utility past due balances (ITT: −13.7%, *p* < 0.001; PP: −15%, *p* = 0.002 versus baseline) which were significantly greater than control group (−3.5%; ITT: *p* = 0.036, PP: *p* = 0.031). Both cohorts reported significant reductions in the rate of shut off notice receipt (ITT: −21.2% *p* = 0.023; PP: −25.3%, *p* = 0.023 versus baseline) which were significantly greater than control (−7.2%; ITT: *p* = 0.017, PP: *p* = 0.007). FSP participants experienced significant reductions in median number of shut off notices (ITT baseline: 3 [2–5] versus 12 month: 3 [1–5], *p* < 0.001; PP baseline: 3 [2–5] versus 12 month: 2 [1–3], *p* < 0.001) which were significantly greater than control (Control baseline 3 [2–5] versus 12 month: 3 [2–5]; ITT: *p* < 0.001; PP: *p* < 0.001).

### Bill Payments

FSP participants reported a significant increase in paying bills on time (ITT: + 20.4%, *p* < 0.001; PP: + 22.1%, *p* < 0.001), which was significantly greater than control (Control: + 5.4%; ITT: *p* = 0.033; PP: *p* = 0.021).

In the ITT analysis, FSP participants reported significant reductions in paying bills with partial payments (−24.4%, *p* < 0.001), ignoring bills (−15.9%, *p* < 0.001), and paying bills with late fees (−21.7%, *p* < 0.001). The reductions were significantly greater than control (−5.42%, *p* = 0.005; −10.2% *p* = 0.085; −4.1% *p* = 0.003, respectively). FSP participants in the ITT analysis also experienced a 20.1% reduction (*p* < 0.001) in overdrawn bank accounts which was significantly greater than control (−7.7%, *p* = 0.046).

In the PP analysis, FSP participants reported significant reductions in paying bills with partial payments (−32.2%, *p* < 0.001), ignoring bills (−23.7%, *p* < 0.001), and paying bills with late fees (−27.4%, *p* < 0.001). The reductions were significantly greater than control (−5.42%, *p* = < 0.001, −10.2% *p* = 0.003, and −4.1% *p* = 0.001, respectively). FSP participants in the PP analysis also reported a significant reduction in the median number of monthly bills with late fees and a 23.2% reduction (p < 0.001) in overdrawn bank accounts which was significantly greater than control, (*p* = 0.033, and p = 0.026, respectively).

### Cash Flow Management

FSP participants reported significant increases in the rates of writing down and prioritizing financial goals (ITT: + 27.6%, *p* < 0.001; PP: + 39%, *p* < 0.001), utilizing a written budget (ITT: + 30.0%, *p* < 0.001; PP: + 41.5%, *p* < 0.001), and reviewing spending (ITT: + 32.7%, *p* < 0.001; PP: + 43.1%, *p* < 0.001). These increases were significantly greater than those of the control group (+ 4%, ITT: P < 0.001 PP: *p* < 0.001; + 10.1% ITT: *p* = 0.004, PP: *p* < 0.001; + 15.9%, ITT: *p* < 0.001, PP: *p* < 0.001).

### Detrimental Financial Behaviors

Payday lenders issue short-term, unsecured cash advances, and are often used to help people out of acute, financially difficult situations. Payday loans typically have much higher interest rates than typical loans, often result in default and can damage credit (National Association of Consumer Advocates, 2026). Use of payday lending significantly decreased in FSP participants (ITT: −11.2%, *p* = 0.002; PP: −12%, *p* = 0.007), however, the reduction was not greater than control (−2.8%, ITT: *p* = 0.057, PP: *p* = 0.056). FSP participants reported significant reductions in sometimes borrowing money (ITT: −23%, *p* < 0.001; PP: −23.7%, *p* < 0.001) which were significantly greater than control (−2.4%, ITT: *p* = 0.003; PP: *p* = 0.006). FSP participants also reported significant increases in those that never maxed out their credit cards (ITT: + 13%, *p* < 0.001; PP: + 12.4%, *p* = 0.002) and a reduction in those that frequently maxed out their credit cards (ITT: −7.4%, *p* = 0.002; PP: −10.5%, *p* = 0.016). Both changes were significantly greater than the control group (Control: + 1.1%, ITT: *p* = 0.015, PP: p = 0.042 and Control: + 4.3%, ITT: p = 0.025, PP: *p* = 0.016, respectively).

## Discussion

A recent meta-analysis of 76 randomized trials assessing financial education demonstrated that programs are effective in improving financial knowledge and downstream financial behaviors (Kaiser et al., 2020). To our knowledge, this is the first randomized, controlled trial to assess the impact of financial education and coaching on ***both*** economic stability and health-related outcomes. Participants who completed the FSP demonstrated significantly reduced financial strain, an increased rate of smoking cessation and a reduction in the avoidance of medical care due to cost completed to the control group (White et al., 2021a, 2021b). In the current analysis, both the PP and ITT analysis found financial education and coaching was associated with significantly improved measures of economic stability. Many financial outcomes were improved in both analyses. Half of the 42 participants in the ITT analysis who were not included in the PP analysis completed some components of the intervention, suggesting that even some amount of FSP has a positive effect on financial outcomes.

The mean salary increase experienced by FSP participants, $5.4 K (23%) ITT and $4.9 K (20%) PP, is much greater than the US average 2.9% merit increase in 2019 (Mercer, 2020). Part of the large increase in salary is attributable to promotions, supported by a significant reduction in clerical positions and a trend towards more administrative positions. In addition, many participants reported starting multiple jobs. Omaha workers report a greater rate of working multiple jobs to make ends meet compared to national data. In 2015, the US average for individuals working multiple jobs was 4.9% (US Bureau of Labor Statistics, 2015). At that same time 14.2% of workers in Omaha, Nebraska held multiple jobs and 7% worked four or more (Nebraska Department of Labor, 2015).

In Nebraska, 21.1% of households are liquid asset poor, meaning they do not have adequate savings to cover basic expenses for three months of job loss (Prosperity Now, 2021). FSP participants significantly increased their rate of savings to 34.5% and 39.6% for the ITT and PP analyses, respectively. It is possible that this increase in savings helped cushion the economic blow from COVID-19. Forty of the 345 participants completed their final study visit after March 2020, during the COVID-19 pandemic. While the impact of COVID-19 on the study results is expected to be evenly distributed between both cohorts, a sub-analysis of this trial found that despite the pandemic, FSP participants experienced fewer job losses and increased their median salaries and ability to save as compared to controls (White et. al., 2021). This suggests that FSP participants were better equipped to adapt to adversity and demonstrate resiliency despite the pandemic.

FSP participants in the PP analysis reported a significant increase in the rate of always contributing to retirement as compared to control while FSP participants in the ITT analysis reported a significant reduction in the rate of always contributing to retirement. Because 24 participants randomized to the FSP in the ITT analysis started, but did not successfully complete programming, retirement contribution may be an effect that requires graduation and subsequent interaction with the coach. When living paycheck to paycheck, retirement savings is often viewed as a ‘luxury’. Also, individuals working multiple part-time jobs often don’t have access to an employer-based retirement savings plan. Participants must also research and choose a plan, which is often overwhelming early in the FSP program. Furthermore, many participants who did not complete the FSP had to withdraw due to sudden life events such as car accidents, issues with children, or job loss. These events could have adversely impacted the ability of participants in the ITT analysis to contribute to retirement.

A SDOH data parameter tracked for the Healthy People 2020 goals was the proportion of households spending more than 30% of income on housing (Healthy People, 2020). In 2007, that rate was 34.6% (Healthy People, 2020). FSP participants experienced significant reductions in the percentage spending ≥ 30% of their gross income on housing. The rate decreased from 46.2% to 29.3% in the ITT analysis and from 44.4% to 30.5% in the PP analysis, both less than the national average in 2007. This can be explained by the fact that as salaries increase, an established benefit of the FSP programming, the housing to income ratio naturally decreases. The effect may also have been compounded, especially later in the study, by falling mortgage interest rates for some participants (Lewis, 2021).

Energy insecurity has been defined as the inability to pay an energy bill, receipt of notice to disconnect, and/or disconnection from an energy service (Memmott et al., 2021). It can lead to use of predatory lending or unsafe alternative heating strategies (Graff & Carley, 2020). In a survey conducted in spring 2020, in households with incomes ≤ 200% of the poverty line, it was found that ~ 25% of low-income households received a shut off notice in the past year. FSP Participants experienced significant increases in utilization of a utility level payment plan. The FSP program works closely with local utility leaders and participants meet one-on-one to discuss level payment and debt resolutions during the classes. Interestingly, the increase in level payment plan use was not significantly greater than the control group. This can be explained by the fact that, anecdotally, at 12-month follow-up visits, multiple control participants reported they had self-enrolled in utility level payment plan because the baseline study survey planted the idea.

FSP participants experienced significant increases in constructive financial behaviors such as writing down and prioritizing financial goals, utilizing a written budget, and reviewing spending. FSP participants also experienced reductions in detrimental financial behaviors such as use of payday lending, borrowing money and maxing out credit cards. As a result of adopting proactive financial behaviors, reducing detrimental behaviors, improving cash flow management, and consolidating debt, FSP participants experienced significant increases in the rate of paying bills on time along with reductions in paying bills with partial payments, ignoring bills, paying bills with late fees and reductions in overdrawn bank accounts. FSP participants learn about the impact of late payments on their credit scores and are trained to advocate for themselves by engaging constructively with creditors to acknowledge and address payment plans, negotiate payment schedules, and resolve disputes. Through focus on monthly cash management, they assess the cumulative impact of bank overdraft fees and apply strategies to reduce these fees in order to reallocate earnings to achieve financial goals.

## Implications for Innovation in Health Services

**Fig. 2:**
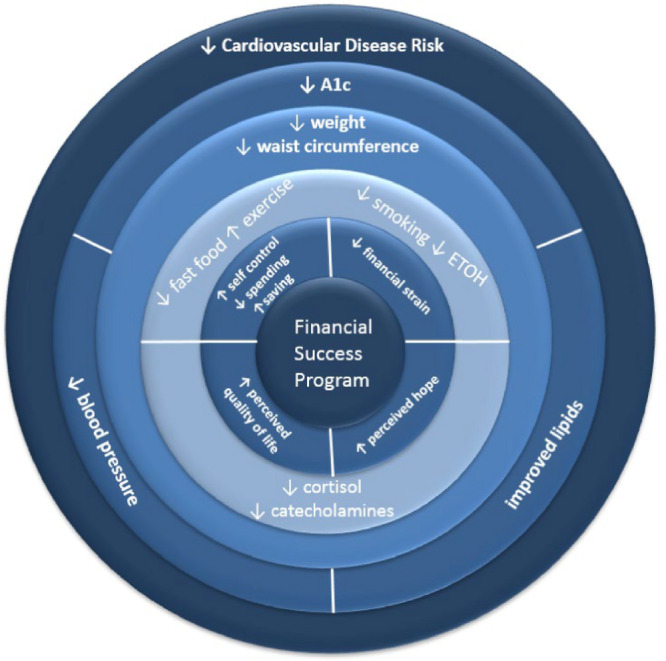
Conceptual model of effects of finances first on health outcomes

This randomized controlled trial was developed to assess the financial and health impact of a novel financial education and coaching program. Figure 2 depicts the conceptual framework for how this intervention can improve the economic stability SDOH and the downstream health benefits, potentially reducing cardiovascular disease risk.

Due to the short duration of this study and the fact that participants study were young and relatively healthy, significant changes in cardiovascular risk factors were not demonstrated in one year (White et al., 2021a, 2021b). However, the impact on upstream financial outcomes and economic strain were realized. If the intervention’s effects on financial stress are durable, a reduced cumulative exposure to financial stress could result in avoidance or reduction of disease in the long-term. In fact, Machado et. al., (2021) recently demonstrated that individuals who experience midlife wealth mobility by at least one quintile, have a significant reduction in cardiovascular outcomes and death after long-term follow up.

The results of this study are promising and support financial education and coaching as a potential health intervention. Follow-up studies are needed to assess for durability and determine the long-term impact of the intervention. Additionally, studies evaluating the impact of the intervention on populations who have diagnosed chronic conditions (hypertension, hyperlipidemia, diabetes) may demonstrate significant reductions in cardiovascular disease risk factors. A study of the FSP as a part of a multi-modal SDOH intervention is currently underway and preliminary data demonstrates promising outcomes including significant blood pressure reductions in a population with type 2 diabetes (Fuji et al., 2025). Evidence of cardiovascular disease risk factor reduction may justify insurance coverage for programs such as the FSP, making them more widely available, and more capable of improving health equity among patients with economic instability and financial stress. Delivery of Financial Education and Coaching is being scaled through an interprofessional medical financial partnership (MFP) model (Prosperity Now, 2021). MFPs are an intersection between financial education and health care systems to collaboratively improve health of low-income communities through financial stress reduction (Bell et al., 2020; Birkenmaier et al., 2024).

## Limitations

From 2011–2017, the FSP had a mean graduation rate of 85%. In this trial it was 63%. Historically, the program runs five times per year with approximately 20 participants per class and has a waiting list. To complete this trial in three years, capacity was increased 1.5-fold and new recruiting strategies, such as radio advertising, were used. Therefore, it is possible that some participants were less motivated than others who may have been on a waiting list. Also, it has been proposed that financial education programs are most successful if they are targeted towards a specific population and the training occurs just before the corresponding event (Hathaway & Khatiwada, 2008). In this situation, single mother households were the target population and the FSP model could have motivated participants to implement change in financial behaviors to improve economic stability. Because the study focused on single mother households, it is also not known how generalizable the results are to other demographic cohorts such as males and married couples. This is currently being studied in the clinic-based MFP.

There were significantly less non-Hispanic White women in the PP analysis (18.3%) as compared to the control group (29.8%). This suggests that a significant proportion of non-Hispanic White women randomized to the FSP did not start or did not graduate. This may be explained by the fact that the majority of FSP alumni are African American and word of mouth is strong in north Omaha. Therefore, it is possible that a higher percentage of the African American women graduated because they were familiar with the program and/or knew a program graduate, making them more open to participating. Systemic racism may have also played a role. White women in Omaha are less likely to have grown up in poverty and may have had access to other resources. Omaha is also very residentially segregated. The location of the programming may have deterred some participants due to north Omaha’s reputation of being unsafe.

## Conclusion

The FSP, an intervention to develop economic stability, was associated with significant improvements in the ability to increase income, earn promotions, increase savings and retirement, and increase constructive financial behaviors while decreasing detrimental financial behaviors in single-mothers with low-income. By mitigating financial stress, women with low-income participating in the FSP increase their capacity to plan ahead and can think more clearly about financial decisions to improve economic stability, and ultimately overall health. **Further evaluation is needed to determine the long-term impact of the intervention as well as the impact of the intervention in populations with existing cardiovascular disease risk factors.**
